# The synergistic effect of depression and moderate chronic kidney disease on the all-cause and cardiovascular disease mortality among adults: a retrospective cohort study

**DOI:** 10.1186/s12882-022-02957-7

**Published:** 2022-10-11

**Authors:** Fanchao Meng, Yanjie Qi, Xu Chen, Xiuping Yan, Huanhuan Huang, Fan He

**Affiliations:** The National Clinical Research Center for Mental Disorder & Beijing Key Laboratory of Mental Disorders, Beijing Anding Hospital, Capital Medical University, Ankang Hutong, No 5, Beijing, 100088 China

**Keywords:** Chronic kidney disease, Depression, Mortality, Cardiovascular disease

## Abstract

**Background:**

The relationship between the coexistence of depression and moderate chronic kidney disease (CKD) and mortality is unclear. We aimed to investigate whether there is a synergistic effect of depression and moderate CKD on the all-cause and cardiovascular disease (CVD) mortality among adults.

**Methods:**

We studied 24,412 participants from the National Health and Nutrition Examination Survey 2005–2014 with a mortality follow-up assessment in 2015. Four groups were created based on depression and CKD status: Group 1, no depression and no CKD; Group 2, depression present and no CKD; Group 3: no depression and CKD present; Group 4: depression present and CKD present. Moderate CKD was defined as an estimated glomerular filtration rate of 15–59 mL/min/1.73 m^2^ (Stage 3–4) or one-time urine albumin-to-creatinine ratio ≥ 30 mg/g. Depression was defined as the 9-item Patient Health Questionnaire score of 10 or more. Cox proportional hazards regression models were used to calculate the multivariate-adjusted hazard ratios (HRs) of death for Group 4 with other groups.

**Results:**

Over a mean follow-up of 5.8 years, 1783 deaths were documented, including 338 deaths from CVD. The HR for all-cause mortality in Group 4 was 3.62 (95% CI: 2.69–4.87), 2.99 (1.92–4.66), and 1.75 (1.29–2.37) when compared with Group 1, 2, and 3, respectively. The HR for CVD mortality in Group 4 was 3.89 (1.68–9.00), 1.90 (0.86–4.21), and 1.97 (1.17–3.32) when compared with Group 1, 2, and 3, respectively.

**Conclusions:**

There might be a synergistic effect of depression and moderate CKD on all-cause mortality. Moreover, depression might increase the risk of CVD mortality in individuals with moderate CKD.

**Supplementary Information:**

The online version contains supplementary material available at 10.1186/s12882-022-02957-7.

## Introduction

Depression is a mood disorder that limits psychosocial functioning and diminishes the quality of life. The lifetime risk of depression is 15–18%, meaning that it is common among the population [1]. There is fairly consistent evidence that depression is associated with increased mortality [2, 3] and poor cardiovascular outcomes [4, 5].

Chronic kidney disease (CKD) is also highly prevalent both in the US and China [6, 7], and multiple studies have documented an increased prevalence of depression in the population with CKD [8]. Previous studies have identified depression as an independent risk factor for death in CKD patients receiving maintenance dialysis [9, 10]. In addition, a meta-analysis, which included 22 studies and 83,381 participants, found that depression consistently increased the risk of all-cause mortality [Risk ratio (RR), 1.59; 95% confidence interval (CI): 1.35–1.87] among adults with CKD [11]. However, the data for individuals with moderate CKD (Stage 3–4) were limited in this study and antidepressant medication use was not adjusted. Moreover, this study did not prove a certain effect of depression on cardiovascular disease (CVD) mortality (RR, 1.88; 95% CI: 0.84–4.19).

Therefore, the relationship between the coexistence of depression and moderate CKD and mortality is worth further exploring. In particular, there is no adequate evidence that proves the effect of depression on the risk of death in people with and without moderate CKD. In this study, we aimed to investigate whether there is a synergistic effect of depression and moderate CKD on the all-cause and CVD mortality among adults.

## Methods

### Data source

We examined the mortality in a nationally representative cohort from the National Health and Nutrition Examination Surveys (NHANES). The NHANES is a series of independent health surveys of the US civilian, noninstitutionalized population conducted by the National Center for Health Statistics (NCHS). Since 1999, NHANES collected data continuously and released datasets in 2-year cycles. In each survey, participants are selected with the use of a complex, stratified, multistage probability-cluster sampling design for in-home interviews and visits to a mobile examination center [12]. We included data from NHANES 2005–2014 as the NHANES used a different rating scale for depression before 2005. The data from NHANES is openly available at https://www.cdc.gov/nchs/nhanes/index.htm. The NCHS approved the study protocols and written informed consent was obtained from each participant [13]. This study followed the STROBE checklist for observational studies.

### Definition of covariates

During the in-home interview, demographic and health-related information was collected by standardized questionnaires [12]. Current smoking was self-reported based on questions about whether participants were currently smoking or not. Current excessive alcohol use was defined as drinking, on average, ≥ 5 drinks/day in the 12 months preceding the survey interview. For the antidepressant medication, participants were asked if they had taken any prescription drug in the past 30 days and antidepressant medication was determined using the second level of drug ingredient categorical codes. A combination of bupropion and naltrexone, which is primarily used for obesity, overweight, and weight-related medical problems, was excluded [14]. History of CVD and cancer was ascertained by self-report and CVD included stroke, congestive heart failure, angina, and myocardial infarction.

Weight and height were measured during the examination and the body mass index (BMI) was defined as weight in kilograms divided by height in meters squared. Blood pressure (BP) was measured and mean BP was calculated as the mean of three readings according to the standardized protocol. Blood samples were collected at the mobile examination center, stored at − 20 °C, and sent to the central laboratories for the measurement of hemoglobin A1c, total cholesterol, and serum creatinine. Urine albumin and creatinine were measured in the same laboratory during the surveys.

Obesity was defined as BMI of 30 or higher; hypertension as systolic BP of 130 mmHg or higher, diastolic BP of 80 mmHg or higher, or the use of antihypertensive medications; diabetes as hemoglobin A1c of 6.5% or higher, or the use of antidiabetic medications; dyslipidemia as total cholesterol of 240 mg/dL or higher, or the use of lipid-lowering medications.

We calculated the estimated glomerular filtration rate (eGFR) based on serum creatinine by using the Chronic Kidney Disease Epidemiology Collaboration equation [15]. Calibrations for the measurements of serum creatinine in different surveys were conducted according to the recommendation of NHANES. Moderate CKD (stage 3–4) was defined as an eGFR of 15–59 mL/min/1.73 m^2^ or a one-time urine albumin-to-creatinine ratio ≥ 30 mg/g. Patients with an eGFR < 15 ml/min/1.73 m^2^, corresponding to CKD stage 5 (end-stage CKD), were excluded.

Depression was assessed using the 9-item Patient Health Questionnaire (PHQ-9) according to the Diagnostic and Statistical Manual of Mental Disorders, fourth edition (DSM-IV). The PHQ-9 is a screening instrument that asks about the frequency of symptoms of depression over the past 2 weeks. We defined major depression as the summed score on the PHQ-9 of 10 points or greater. The use of a PHQ-9 score of 10 points has been proven to provide a favorable balance between sensitivity and specificity [16].

### Ascertain of mortality

Mortality data were retrospectively ascertained by linkage to the National Death Index through December 31, 2015. The International Classification of Diseases-10 was used to determine disease-specific mortality. According to the Codebook for the 2015 Public-Use Linked Mortality File, CVD mortality included heart disease mortality and stroke mortality [17].

### Statistical analysis

To account for oversampled population, appropriate 10-year sampling weights were constructed according to the NHANES recommendation. All statistical analysis was conducted in R version 4.1.0 with the “Survey” package after accounting for the complex sampling design. All statistical tests were 2-sided, and *P* < 0.05 was considered statistically significant. We restricted our participants to non-pregnant adults aged 18 years or older. Continuous variables were expressed as means (standard error) and categorical variables as percentages (standard error) in the four different groups. Group 1 was defined as participants without depression and without moderate CKD, Group 2 as participants with depression and without moderate CKD. Group 3 as participants without depression and with moderate CKD, and Group 4 as participants with both depression and moderate CKD. Mortality was expressed as events per 1000 person-years of follow-up. The number of person-years of follow-up time was calculated from the time of entry into the study until the date of death or the termination day of follow-up. Life expectancy and survival times for four groups were estimated by the Kaplan-Meier product-limit method.

Cox proportional hazards regression models were used to calculate multivariate-adjusted hazard ratios (HRs) of death across different groups. We calculated native HR in model 0 without adjusting for any confounders. We also calculated adjusted HRs in three different models. Model 1 was adjusted for age, gender, race, and education. Model 2 was further adjusted for obesity, diabetes, hypertension, dyslipidemia, history of cardiovascular disease, and history of cancers based on Model 1. Model 3 was further adjusted for current smoke, current excessive alcohol use, antidepressant medication, eGFR, and albumin-to-creatinine ratio based on model 2. Three pairs of post hoc comparisons with Bonferroni correction were conducted after the main analysis to test the difference of death in Group 4 with death in the other three groups [18]. A *P*-value less than 0.017 was considered statistically significant in the post hoc analysis.

We conducted several sensitivity analyses to test the robustness of the results. First, to account for the difference in the definition of CKD, we redefined CKD as an eGFR of 15 to 59 mL/min/1.73 m^2^ alone without considering the urine albumin-to-creatinine ratio. Second, to account for the potential effects of age on the outcomes, we excluded young adults in the analysis and restricted our participants to those aged 45 years or older. Third, to account for the interaction between depressive symptoms and functional impairment, we compared mortality rates among four groups of participants based on the presence or absence of functional impairment and moderate CKD. In these sensitivity analyses, we only compared the all-cause mortality in different groups owing to the small number of CVD death events.

## Results

The baseline characteristics of participants are presented in Table 1. Our final data set comprised 24,412 participants. In general, compared to those without depression, depressed participants with or without CKD were younger, less likely to be male and non-Hispanic white, received less education, more likely to be current smokers and current excessive alcohol and antidepressant users, more likely to be obese, and more likely to have a history of hypertension, diabetes, dyslipidemia, CVD, and cancer.

**Table 1:** Baseline characteristics of participants by presence or absence of CKD and depression, NHANES 2005–2014

	Group 1	Group 2	Group 3	Group 4
	Without CKD and without depression	Without CKD and with depression	With CKD and without depression	With CKD and with depression
Number of Participants	18,334	1695	3928	455
Age, year	44.3 (0.3)	44.1 (0.5)	60.2 (0.5)	56.2 (0.9)
Male, %	52.0 (0.4)	36.4 (1.6)	43.7 (1.1)	32.1 (2.6)
Race/ethnicity, %				
Mexican American	13.5 (1.0)	16.1 (1.6)	11.2 (1.0)	16.0 (2.1)
Non-Hispanic white	70.0 (1.5)	64.8 (2.2)	72.2 (1.5)	61.5 (3.0)
Non-Hispanic black	10.1 (0.8)	13.2 (1.2)	11.6 (0.9)	18.2 (2.1)
Other	6.5 (0.4)	5.9 (0.7)	5.0 (0.5)	4.3 (1.1)
Education, %				
Less than high school	15.7 (0.7)	27.2 (1.6)	23.1 (0.9)	35.5 (3.1)
High school graduate	22.6 (0.6)	27.2 (1.5)	25.6 (1.2)	25.9 (2.9)
Some college	31.6 (0.6)	33.1 (1.5)	29.4 (1.0)	32.0 (3.0)
College graduate or higher	30.1 (1.0)	12.5 (1.4)	22.0 (1.2)	6.6 (1.8)
Current smoke, %	20.4 (0.6)	40.7 (1.8)	15.8 (0.7)	33.9 (3.2)
Current excessive alcohol use, %	11.8 (0.4)	15.2 (1.0)	6.2 (0.6)	9.0 (1.5)
Antidepressant medication, %	19.7 (0.6)	48.6 (2.1)	18.2 (0.9)	45.0 (3.1)
Obesity, %	33.1 (0.6)	44.3 (1.7)	41.7 (1.1)	55.6 (3.2)
Hypertension, %	40.1 (0.6)	44.8 (1.5)	72.5 (1.0)	74.9 (2.8)
Diabetes, %	6.8 (0.2)	11.7 (1.2)	26.8 (1.0)	39.8 (2.5)
Dyslipidemia, %	24.8 (0.5)	29.0 (1.6)	45.8 (1.2)	48.2 (3.2)
History of CVD, %	5.4 (0.2)	12.6 (1.0)	23.2 (1.0)	36.4 (3.1)
History of Cancer, %	8.3 (0.3)	11.7 (1.1)	17.1 (0.7)	14.4 (2.2)
eGFR, mL/min/1.73 m^2^	98.1 (0.3)	99.3 (0.6)	72.8 (0.7)	76.0 (1.8)
Albumin-to-creatinine ratio, mg/g	7.6 (0.1)	8.3 (0.2)	155.0 (10.1)	269.9 (35.4)

*CKD* chronic kidney disease, *CVD* cardiovascular disease, *eGFR* estimated glomerular filtration rate, *NHANES* National Health and Nutrition Examination Surveys

### All-cause mortality

**Fig. 1:**
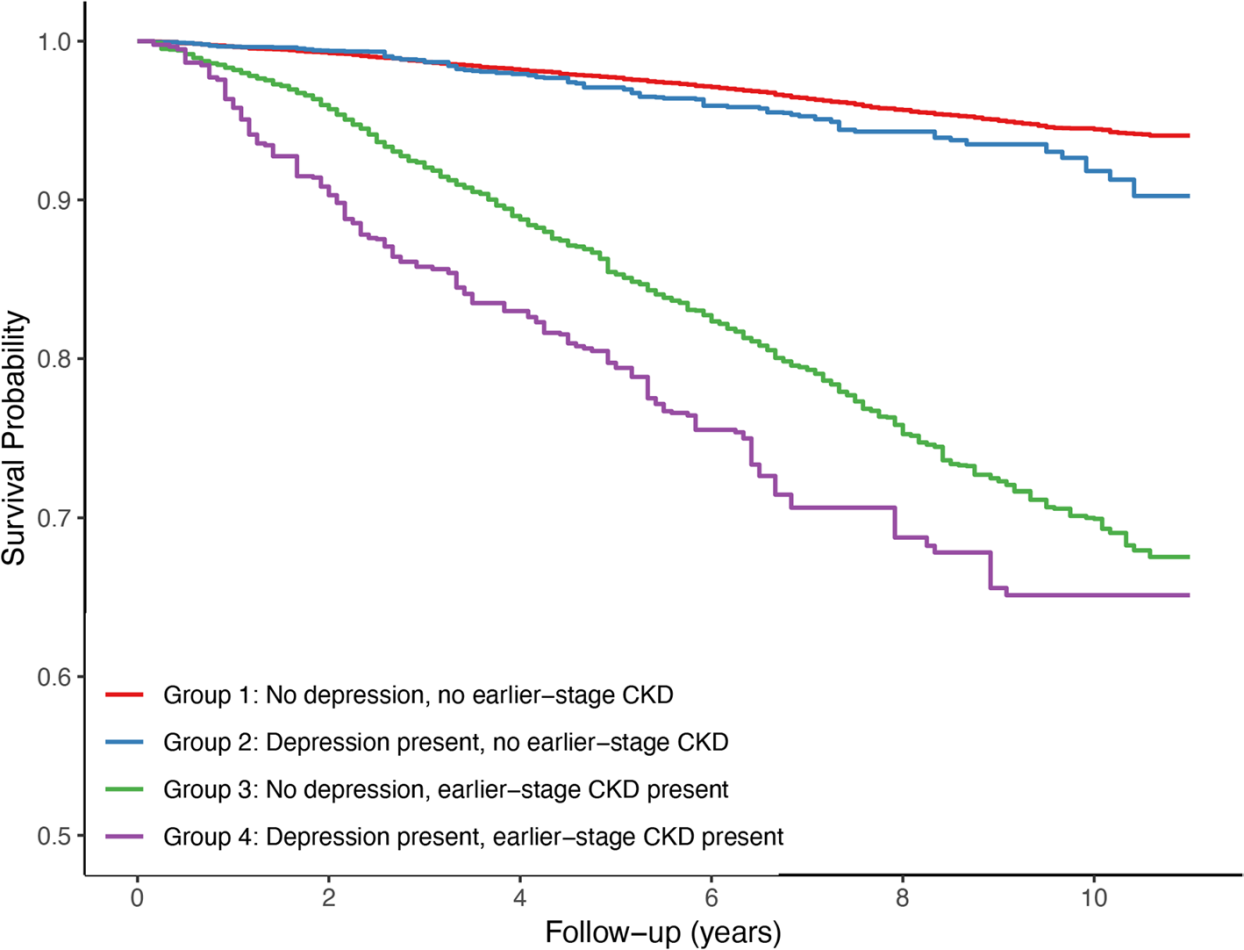
Estimated probability of survival (all-cause mortality) according to depression and moderate CKD status. CKD: chronic kidney disease

Table 2 presents all-cause and CVD mortality rates and adjusted HRs of death. The mean follow-up period was 5.8 years and a total of 1783 deaths from all-cause were recorded. The all-cause mortality rate was 5.2, 6.9, 32.8, and 46.6 per 1000 person-years in Group 1, Group 2, Group 3, and Group 4, respectively. Figure 1 shows the Kaplan-Meier survival curves of unadjusted all-cause mortality for four different groups. In model 1, model 2, and model 3, when compared with Group 1, adjusted HRs for deaths from all-cause were significantly higher in Group 2 and Group 3 but were highest in Group 4.

**Table 2:** The hazard ratio of all-cause and CVD mortality according to the presence of CKD and depression

	Group 1	Group 2	Group 3	Group 4
	Without CKD and without depression	Without CKD and with depression	With CKD and without depression	With CKD and with depression
Number of Participants	18,334	1695	3928	455
Follow-up (person-years)	106,846	9565	21,100	2125
All-cause mortality				
Events (n)	785	79	809	110
Mortality rate (95% CI)#	5.2 (4.7–5.8)	6.9 (5.2–8.6)	32.8 (30.0–35.7)	46.6 (36.3–56.9)
Hazard ratio (95% CI)				
Model 0	1.00 (reference)	1.33 (1.00–1.78)	6.36 (5.61–7.22)	9.26 (7.19–11.93)
Model 1	1.00 (reference)	1.50 (1.14–1.98)	2.16 (1.88–2.48)	4.38 (3.45–5.56) †
Model 2	1.00 (reference)	1.46 (1.10–1.93)	1.84 (1.59–2.12)	3.46 (2.63–4.54) †
Model 3	1.00 (reference)	1.43 (1.06–1.94)	1.86 (1.52–2.27)	3.24 (2.37–4.42) †
CVD mortality				
Events (n)	125	15	172	26
Mortality rate (95% CI)	0.8 (0.6 to 1.0)	1.5 (0.6–2.5)	6.6 (5.3–7.9)	10.3 (5.4–15.2)
Hazard ratio (95% CI)				
Model 0	1.00 (reference)	1.96 (0.97–3.95)	8.53 (6.38–11.40)	13.5 (7.88–23.00)
Model 1	1.00 (reference)	2.51 (1.25–5.04)	2.33 (1.71–3.19)	5.72 (3.39–9.67) ‡
Model 2	1.00 (reference)	2.35 (1.15–4.78)	1.87 (1.36–2.58)	3.91 (2.21–6.93) ‡
Model 3	1.00 (reference)	2.33 (1.12–4.83)	1.89 (1.31–2.72)	3.43 (1.82–6.45) ‡

*CI* confidence interval, *CKD* chronic kidney disease, *CVD* cardiovascular disease

† In the post hoc comparison, when compared with Group 1, Group 4 had an adjusted HR for all-cause mortality rate of 4.18 (95% CI: 3.26–5.35; *P* < 0.001), 3.44 (95% CI: 2.61–4.53; *P* < 0.001), and 3.62 (95% CI: 2.69–4.87; *P* < 0.001) in model 1, model 2, and model 3, respectively; Compared with Group 2, Group 4 had an adjusted HR for all-cause mortality rate of 3.37 (95% CI: 2.30–4.92; *P* < 0.001), 2.76 (95% CI: 1.84–4.14; *P* < 0.001), and 2.99 (95% CI: 1.92–4.66; *P* < 0.001) in model 1, model 2, and model 3, respectively; Compared with Group 3, Group 4 had an adjusted HR for all-cause mortality rate of 2.03 (95% CI: 1.62–2.55; *P* < 0.001), 1.87 (95% CI: 1.43–2.45; *P* < 0.001), and 1.75 (95% CI: 1.29–2.37; *P* < 0.001) in model 1, model 2, and model 3, respectively

‡ In the post hoc comparison, when compared with Group 1, Group 4 had an adjusted HR for CVD mortality rate of 5.64 (95% CI: 3.43–9.25; *P* < 0.001), 3.92 (95% CI: 2.21–6.94; *P* < 0.001), and 3.89 (95% CI: 1.68–9.00; *P* = 0.002), in model 1, model 2, and model 3, respectively; Compared with Group 2, Group 4 had an adjusted HR for CVD mortality rate of 2.64 (95% CI: 1.19–5.85; *P* = 0.0173), 2.01 (95% CI: 0.93–4.34; *P* = 0.074), and 1.90 (95% CI: 0.86–4.21; *P* = 0.112) in model 1, model 2, and model 3, respectively; Compared with Group 3, Group 4 had an adjusted HR for CVD mortality rate of 2.25 (95% CI: 1.44–3.50; *P* < 0.001), 2.00 (95% CI: 1.19–3.35; *P* = 0.009), and 1.97 (95% CI: 1.17–3.32; *P* = 0.011) in model 1, model 2, and model 3, respectively

In the post hoc comparison, adjusted HRs for deaths from all-cause were significantly higher in Group 4 when compared with Group 1 in model 1 (HR, 4.18; 95% CI, 3.26–5.35; *P* < 0.001), model 2 (HR, 3.44; 95% CI, 2.61–4.53; *P* < 0.001) and model 3 (HR, 3.62; 95% CI: 2.69–4.87; *P* < 0.001). The adjusted HRs for deaths from all-cause in Group 4 were all significantly higher when compared with Group 2 and Group 3 in model 1, model 2, and model 3.

In the sensitivity analysis with the definition of CKD as an eGFR of 15 to 59 mL/min/1.73 m^2^ alone, Group 4 had significantly higher adjusted HRs for all-cause mortality rate when compared with Group 1, Group 2, and Group 3 in model 1 and model 2 (Supplemental Table 1). In model 3, Group 4 had a higher adjusted HR (1.61; 95% CI: 1.02–2.56) when compared with Group 3. However, the *P*-value (0.042) was larger than 0.017 and should not be considered statistically significant in the post hoc analysis. In another two sensitivity analyses, Group 4 had significantly higher adjusted HRs for all-cause mortality rate when compared with Group 1, Group 2, and Group 3 in model 1, model 2, and model 3 (Supplemental Table 1). The Kaplan-Meier survival curves of unadjusted all-cause mortality for four different groups in the sensitivity analyses were presented in Supplemental Fig. 1, 2, and 3.

### CVD mortality

**Fig. 2:**
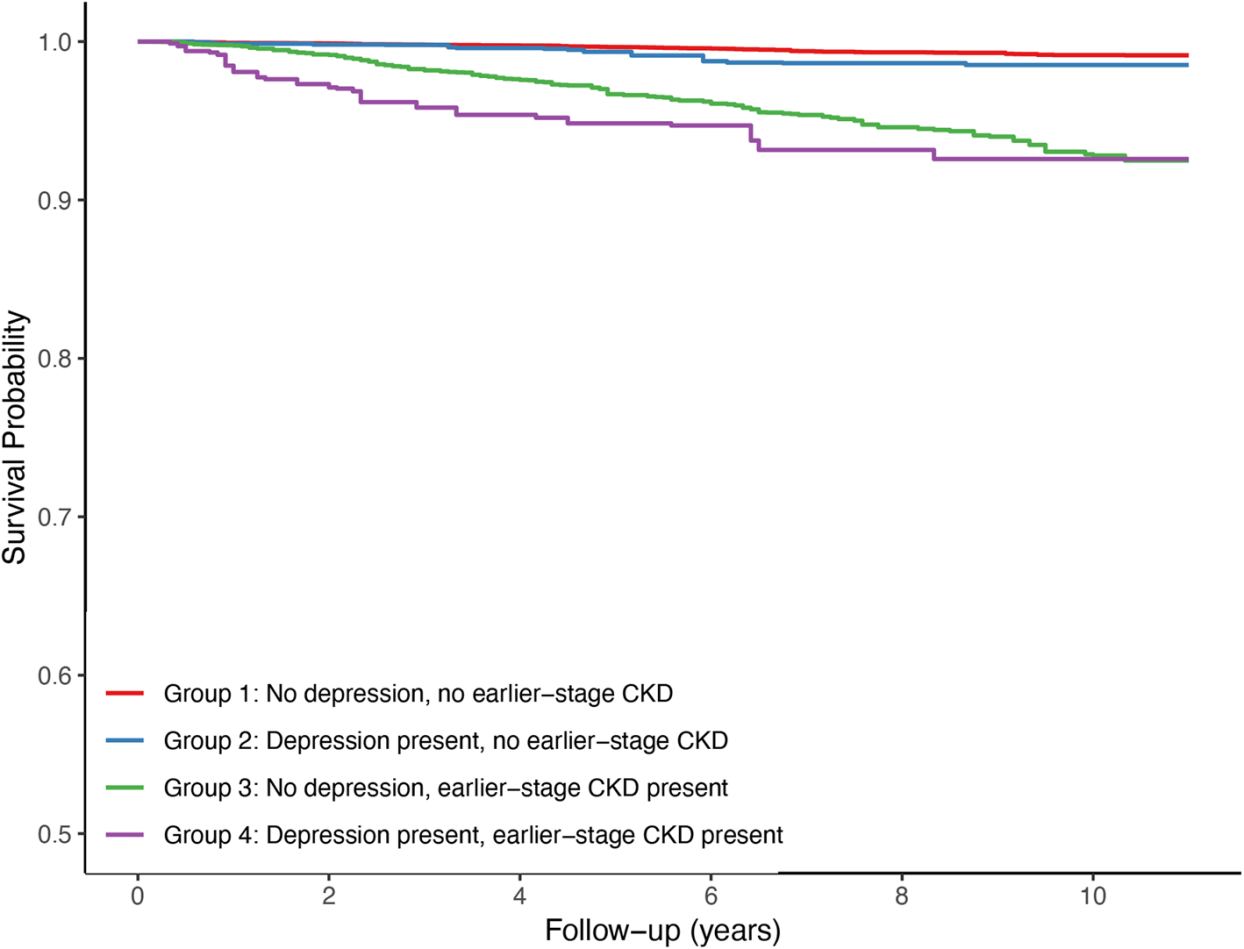
Estimated probability of survival (cardiovascular disease mortality) according to depression and moderate CKD status. CKD: chronic kidney disease

A total of 338 individuals died from CVD. The CVD mortality rate was 0.8, 1.5, 6.6, and 10.3 per 1000 person-years in Group 1, Group 2, Group 3, and Group 4, respectively. Figure 2 shows the Kaplan-Meier survival curves of unadjusted CVD mortality for four different groups. In model 1, model 2, and model 3, adjusted HRs for deaths from CVD were significantly higher in Group 2 and Group 3 but were highest in Group 4. In model 1, model 2, and model 3, when compared with Group 1, adjusted HRs for deaths from CVD were significantly higher in Group 2 and Group 3 but were highest in Group 4.

In the post hoc comparison, adjusted HRs for deaths from CVD were significantly higher in Group 4 when compared with Group 1 in model 1 (HR, 5.64; 95% CI, 3.43–9.25; *P* < 0.001), model 2 (HR, 3.92; 95% CI, 2.21–6.94; *P* < 0.001) and model 3 (HR, 3.89; 95% CI: 1.68–9.00; *P* = 0.002). The adjusted HRs for deaths from CVD in Group 4 were also significantly higher when compared with Group 3 in model 1 (HR, 2.25; 95% CI, 1.44–3.50; *P* < 0.001), model 2 (HR, 2.00; 95% CI, 1.19–3.35; *P* = 0.009), and model 3 (HR, 1.97; 95% CI, 1.17–3.32; *P* = 0.011). However, the adjusted HRs for deaths from CVD in Group 4 were not statistically significant higher when compared with Group 2 in model 1 (HR, 2.64; 95% CI: 1.19–5.85; *P* = 0.0173), model 2 (HR, 2.01; 95% CI: 0.93–4.34; *P* = 0.074), and model 3 (HR, 1.90; 95% CI: 0.86–4.21; *P* = 0.112).

## Discussion

In this study, we demonstrated that there might be a synergistic effect of depression and moderate CKD on all-cause mortality. After adjusting for clinically potential cofounders, people with both depression and moderate CKD are 1.75 times as likely to die from all-cause when compared with those with moderate CKD alone, 2.99 times as likely to die from all-cause compared with those with depression alone, and 3.62 times as likely to die from all-cause compared with those without either depression and moderate CKD. In addition, depression might increase the risk of CVD mortality in individuals with moderate CKD as those with both depression and moderate CKD are 1.97 times as likely to die from CVD mortality compared to those with moderate CKD alone.

Numerous studies of end-stage renal disease patients receiving maintenance dialysis reported an association between depression and increased risk of poor outcomes, including death [9, 10, 19–21]. However, the number of studies evaluating the relationship between depression in people with moderate CKD and mortality is limited. Hedayati et al. explored the association between major depressive episodes in 267 participants with CKD (stage 2–5 and who were not receiving dialysis) and a composite of death, dialysis initiation, or hospitalization in a prospective cohort study with one-year follow-up [22]. They found that the presence of depression was associated with an increased risk of a composite of events in CKD patients. However, in this study, the relationship between depression and death was not significant, which might be due to the small number of participants. Even though depression in moderate CKD was proved to be an independent predictor of adverse clinical outcomes in these studies, its relationship with mortality was not clearly defined. To our knowledge, the current study was the first that reports a significant synergistic effect of depression and moderate CKD on mortality among adults. This effect was robust and independent of demographic factors, medical comorbidities, and especially the antidepressant medication.

There is accumulating evidence that patients with CKD exhibit a pronounced risk for CVD and that it is associated with increased CVD mortality [23, 24]. Similarly, considerable evidence proves that depression is associated with a significantly increased risk of CVD mortality [4, 25–27]. The results of our study were consistent with previous findings. Compared with people without moderate CKD and without depression, those with moderate CKD alone had 1.89-fold increased CVD mortality and those with depression alone had 2.33-fold increased CVD mortality. Moreover, even though individuals with both depression and moderate CKD did not have a significantly higher risk of CVD mortality compared with those with depression alone after adjusting for all the potential confounders, they had a 1.97-fold increased CVD mortality when compared with those with moderate CKD alone. These findings suggested that depression might be a stronger predictor of CVD mortality than moderate CKD in this cohort and depression accounts for most of the increased risk of CVD mortality in individuals with both depression and moderate CKD.

The underlying mechanisms that depression increases mortality are unclear. Suicide is a clear mechanism through which depression increases mortality. Another plausible mechanism is nonadherence to medical advice, which is consequently associated with decreased survival [28]. Furthermore, persistent depression would worsen physical health over time and psychological distress with subsequent neurohormonal changes is thought to increase susceptibility to disease [29]. However, it remains unclear whether depression itself has a direct impact on the development of mortality in CKD or whether depression is just a surrogate marker for comorbidity and CVD severity [9]. Our study found that after adjusting for comorbidity and other risk factors, the association between depression and mortality remains significant even though the HR decreased, indicating that depression might have both direct and indirect effects on mortality in patients with CKD.

The limitations of this study should be noted. First and most important, the measurement of serum creatinine and urine albuminuria was only available at a single point in time in NHANES and it might lead to the misclassification of CKD status. However, the misclassification should be non-differential across survey years and it is not likely to bias our analysis. Second, we did not include some other factors such as inflammation, morbidity, or mineral bone disease as confounders since they were not complete or available in the NHANES. Notwithstanding, this is not unprecedented and previous similar studies also did not include them as confounders [22, 30, 31]. Third, we used self-reported questionnaires, making data susceptible to recall and information biases. However, self-reported information on depression and CVD risk factors has been widely used in epidemiological studies and the results are reliable. Fourth, the current study was observational and it is unlikely to determine whether depression is causally related to excessive mortality risk in individuals with moderate CKD. Last, the number of CVD events in the follow-up was relatively small and we are not able to evaluate the impact of depression on CVD mortality in the sensitivity analyses. Thus, the interpretation of the significant association between depression and CVD mortality in individuals with moderate CKD should be with caution.

Despite these potential limitations, the results of this study remain meaningful. There might be a synergistic effect of depression and moderate CKD on all-cause mortality. Moreover, depression might increase the risk of CVD mortality in individuals with moderate CKD.

## Supplementary Information

**Additional file 1: Supplemental Table 1.** Hazard ratio of all-cause mortality in the sensitivity analysis. **Supplemental Fig. 1.** Estimated probability of survival (all-cause mortality) according to depression and earlier-stage CKD status in sensitivity analysis with the definition of CKD as eGFR of 15–59 mL/min/1.73 m^2^ alone. CKD: chronic kidney disease; eGFR: estimated glomerular filtration rate. **Supplemental Fig. 2.** Estimated probability of survival (all-cause mortality) according to depression and earlier-stage CKD status in sensitivity analysis with participants aged 45 years or older. CKD: chronic kidney disease. **Supplemental Fig. 3.** Estimated probability of survival (all-cause mortality) according to functional impairment and earlier-stage CKD status. CKD: chronic kidney disease.

## Data Availability

The data is from the National Health and Nutrition Examination Survey and is openly available at https://www.cdc.gov/nchs/nhanes/index.htm.

## Abbreviations

BP: Blood pressure
BMI: Body mass index
CI: Confidence interval
CKD: Chronic kidney disease
CVD: Cerebrovascular disease
DSM-IV: Diagnostic and Statistical Manual of Mental Disorders, fourth edition
eGFR: Estimated glomerular filtration rate
HR: Hazard ratio
RR: Risk ratio
NCHS: National Center for Health Statistics
NHANES: National Health and Nutrition Examination Surveys
PHQ-9: 9-item Patient Health Questionnaire
