# Supplementary material for: The synergistic effect of depression and moderate chronic kidney disease on the all-cause and cardiovascular disease mortality among adults: a retrospective cohort study
Source: BMC Nephrol. 2022 Oct 11;23:330. doi: 10.1186/s12882-022-02957-7 (PMC9554995; doi:10.1186/s12882-022-02957-7)

| Supplemental Table 1. Hazard ratio of all-cause mortality in the sensitivity analysis | | | | |
| --- | --- | --- | --- | --- |
| **CKD definition by eGFR alone** | | | | |
|  | Group 1 | Group 2 | Group 3 | Group 4 |
|  | Without CKD and without depression | Without CKD and with depression | With CKD and without depression | With CKD and with depression |
| Number of Participants | 20266 | 1952 | 1834 | 170 |
| Follow-up (person-years) | 117758 | 10865 | 9420 | 697 |
| All-cause mortality |  |  |  |  |
| Events (n) | 1025 | 126 | 519 | 52 |
| Mortality rate (95% CI) | 6.3 (5.7-6.9) | 9.4 (7.5-11.4) | 46.8 (41.8-51.7) | 70.8 (46.4-95.2) |
| Hazard ratio (95% CI) |  |  |  |  |
| Model 0 | 1.00 (reference) | 1.52 (1.20-1.92) | 7.66 (6.78-8.64) | 12.14 (8.30-17.76) |
| Model 1 | 1.00 (reference) | 1.73 (1.39-2.14) | 1.68 (1.47-1.91) | 3.33 (2.30-4.82)† |
| Model 2 | 1.00 (reference) | 1.59 (1.27-2.00) | 1.45 (1.27-1.65) | 2.74 (1.81-4.14) † |
| Model 3 | 1.00 (reference) | 1.54 (1.19-2.00) | 1.33 (1.08-1.64) | 2.17 (1.46-3.24) † |
| **Participants aged 45 years or older** | | | | |
|  | Group 1 | Group 2 | Group 3 | Group 4 |
|  | Without CKD and without depression | Without CKD and with depression | With CKD and without depression | With CKD and with depression |
| Number of Participants | 8974 | 897 | 3221 | 352 |
| Follow-up (person-years) | 51268 | 4965 | 16876 | 1536 |
| All-cause mortality |  |  |  |  |
| Events (n) | 664 | 66 | 794 | 99 |
| Mortality rate (95% CI) | 9.2 (8.1-10.2) | 10.9 (8.2-13.7) | 41.6 (38.1-45.1) | 59.9 (44.7-75.2) |
| Hazard ratio (95% CI) |  |  |  |  |
| Model 0 | 1.00 (reference) | 1.20 (0.90-1.60) | 4.65 (4.01-5.40) | 6.99 (5.17-9.46) |
| Model 1 | 1.00 (reference) | 1.58 (1.20-2.09) | 2.01 (1.73-2.34) | 4.08 (3.13-5.34)‡ |
| Model 2 | 1.00 (reference) | 1.52 (1.15-2.00) | 1.72 (1.48-2.00) | 3.29 (2.43-4.47)‡ |
| Model 3 | 1.00 (reference) | 1.45 (1.08-1.96) | 1.72 (1.39-2.13) | 2.96 (2.07-4.23)‡ |
| **Presence of CKD and Functional Impairment** | | | | |
|  | Group 1 | Group 2 | Group 3 | Group 4 |
|  | Without CKD and without functional Impairment | Without CKD and with functional Impairment | With CKD and without functional Impairment | With CKD and with functional Impairment |
| Number of Participants | 9594 | 3635 | 2111 | 895 |
| Follow-up (person-years) | 55846 | 20996 | 11198 | 4623 |
| All-cause mortality |  |  |  |  |
| Events (n) | 376 | 167 | 429 | 214 |
| Mortality rate (95% CI) | 4.8 (4.1-5.6) | 6.3 (5.2-7.5) | 32.1 (27.5-36.7) | 43.6 (37.1-50.0) |
| Hazard ratio (95% CI) |  |  |  |  |
| Model 0 | 1.00 (reference) | 1.32 (1.05-1.65) | 6.76 (5.49-8.32) | 9.18 (7.47-11.30) |
| Model 1 | 1.00 (reference) | 1.46 (1.17-1.83) | 2.39 (1.91-2.99) | 3.88 (3.20-4.71)§ |
| Model 2 | 1.00 (reference) | 1.41 (1.12-1.78) | 2.04 (1.64-2.55) | 3.02 (2.47-3.70)§ |
| Model 3 | 1.00 (reference) | 1.28 (0.96-1.70) | 1.93 (1.45-2.58) | 2.99 (2.31-3.87)§ |
| CI: confidence interval; CKD: chronic kidney disease; CVD: cardiovascular disease; eGFR: estimated glomerular filtration rate.  † In the post hoc comparison, when compared with Group 1, Group 4 had an adjusted HR for all-cause mortality rate of 3.21 (95% CI: 2.21-4.67; P<0.001), 2.68 (95% CI: 1.78-4.04; P<0.001), and 3.20 (95% CI: 1.98-5.18; P<0.001) in model 1, model 2, and model 3, respectively; Compared with Group 2, Group 4 had an adjusted HR for all-cause mortality rate of 2.15 (95% CI: 1.36-3.39; P=0.001), 1.96 (95% CI: 1.19-3.24; P=0.009), and 2.61 (95% CI: 1.25-5.44; P=0.011) in model 1, model 2, and model 3, respectively; Compared with Group 3, Group 4 had an adjusted HR for all-cause mortality rate of 2.05 (95% CI: 1.41-3.00; P<0.001), 1.97 (95% CI: 1.27-3.07; P=0.003), and 1.61 (95% CI: 1.02-2.56; P=0.042) in model 1, model 2, and model 3, respectively.  ‡ In the post hoc comparison, when compared with Group 1, Group 4 had an adjusted HR for CVD mortality rate of 3.86 (95% CI: 2.92-5.10; P<0.001), 3.28 (95% CI: 2.40-4.49; P<0.001), and 3.38 (95% CI: 2.42-4.73; P<0.001), in model 1, model 2, and model 3, respectively; Compared with Group 2, Group 4 had an adjusted HR for CVD mortality rate of 3.13 (95% CI: 2.04-4.82; P<0.001), 2.80 (95% CI: 1.61-4.86; P<0.001), and 2.80 (95% CI: 1.75-4.48; P<0.001) in model 1, model 2, and model 3, respectively; Compared with Group 3, Group 4 had an adjusted HR for CVD mortality rate of 1.95 (95% CI: 1.52-2.49; P<0.001), 1.83 (95% CI: 1.37-2.45; P<0.001), and 1.71 (95% CI: 1.25-2.34; P<0.001) in model 1, model 2, and model 3, respectively.  §In the post hoc comparison, when compared with Group 1, Group 4 had an adjusted HR for CVD mortality rate of 3.86 (95% CI: 3.18-4.69; P<0.001), 3.04 (95% CI: 2.48-3.74; P<0.001), and 2.68 (95% CI: 2.01-3.58; P<0.001), in model 1, model 2, and model 3, respectively; Compared with Group 2, Group 4 had an adjusted HR for CVD mortality rate of 2.70 (95% CI: 2.11-3.44; P<0.001), 2.17 (95% CI: 1.63-2.90; P<0.001), and 2.29 (95% CI: 1.48-3.52; P<0.001) in model 1, model 2, and model 3, respectively; Compared with Group 3, Group 4 had an adjusted HR for CVD mortality rate of 1.61 (95% CI: 1.33-1.95; P<0.001), 1.47 (95% CI: 1.19-1.83; P<0.001), and 1.59 (95% CI: 1.25-2.02; P<0.001) in model 1, model 2, and model 3, respectively. | | | | |

Supplemental Figure 1: Estimated probability of survival (all-cause mortality) according to depression and earlier-stage CKD status in sensitivity analysis with the definition of CKD as eGFR of 15-59 mL/min/1.73 m^2^ alone. CKD: chronic kidney disease; eGFR: estimated glomerular filtration rate.


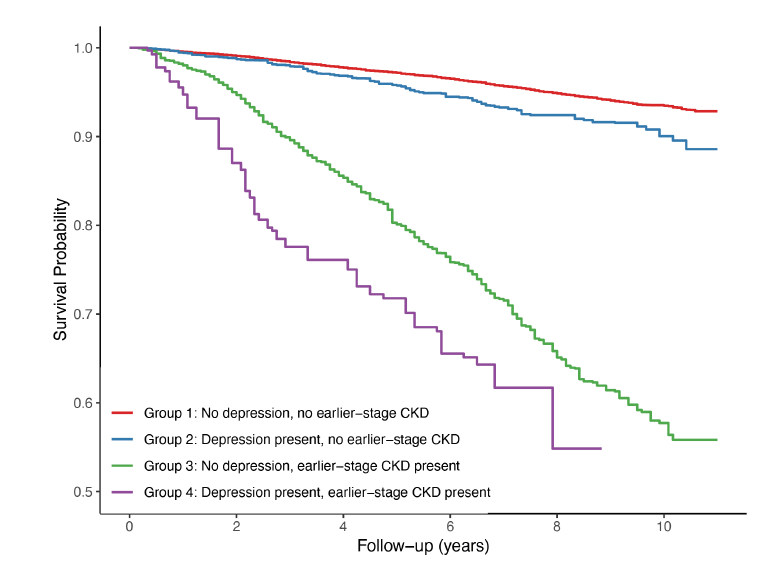


Supplemental Figure 2: Estimated probability of survival (all-cause mortality) according to depression and earlier-stage CKD status in sensitivity analysis with participants aged 45 years or older. CKD: chronic kidney disease.


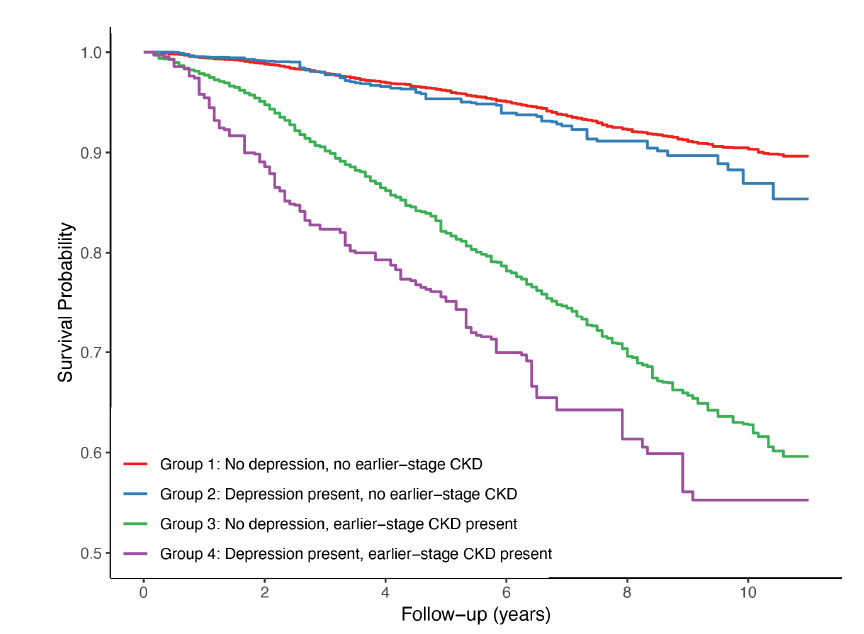


Supplemental Figure 3: Estimated probability of survival (all-cause mortality) according to functional impairment and earlier-stage CKD status. CKD: chronic kidney disease.


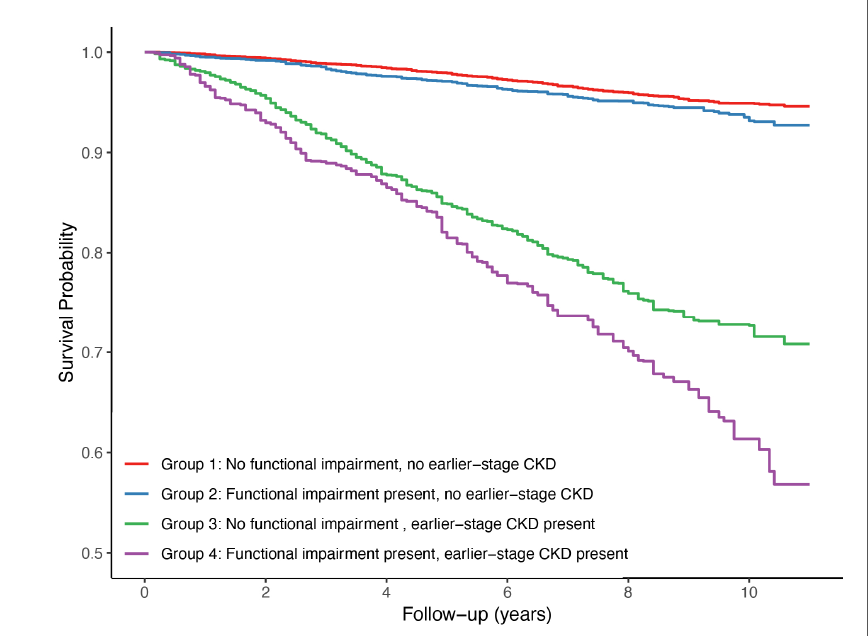

Supplement: Supplementary file 1 — Additional file 1: Supplemental Table 1. Hazard ratio of all-cause mortality in the sensitivity analysis. Supplemental Fig. 1. Estimated probability of survival (all-cause mortality) according to depression and earlier-stage CKD status in sensitivity analysis with the definition of CKD as eGFR of 15–59 mL/min/1.73 m2 alone. CKD: chronic kidney disease; eGFR: estimated glomerular filtration rate. Supplemental Fig. 2. Estimated probability of survival (all-cause mortality) according to depression and earlier-stage CKD status in sensitivity analysis with participants aged 45 years or older. CKD: chronic kidney disease. Supplemental Fig. 3. Estimated probability of survival (all-cause mortality) according to functional impairment and earlier-stage CKD status. CKD: chronic kidney disease. [file 12882_2022_2957_MOESM1_ESM.docx]
